# Enhancing motivation for initiation and continuation of physical activity in older adults using sensory augmentation devices: a preliminary pre–post study

**DOI:** 10.3389/fspor.2025.1512002

**Published:** 2025-08-22

**Authors:** Yusuke Sakaue, Yurie Kondo, Masaaki Makikawa, Naruhiro Shiozawa, Motoyuki Iemitsu, Tetsuo Yoshimoto, Shima Okada

**Affiliations:** ^1^ Ritsumeikan Global Innovation Research Organization, Ritsumeikan University, Kusatsu, Shiga, Japan; ^2^ Graduate School of Science and Engineering, Ritsumeikan University, Kusatsu, Shiga, Japan; ^3^ Research Organization of Science and Technology, Ritsumeikan University, Kusatsu, Shiga, Japan; ^4^ Department of Sport and Health Science, College of Sport and Health Science, Ritsumeikan University, Kusatsu, Shiga, Japan; ^5^ Department of Business Administration, College of Business Administration, Ritsumeikan University, Ibaraki, Osaka, Japan; ^6^ Department of Robotics, College of Science and Engineering, Ritsumeikan University, Kusatsu, Shiga, Japan

**Keywords:** physical activity, intrinsic capability, enjoyment, sensory augmentation, self-efficacy

## Abstract

Improvement of physical and cognitive capabilities through physical activity (PA) contributes to well-being in older age. Unfortunately, many older adults lack sufficient PA, due to reasons such as PA not being enjoyable for them. As a result, older adults are unable to maintain their motivation for the initiation and continuation of PA. In our previous works, a boxing glove-type sensory augmentation system was developed to solve this problem by inducing enjoyment during PA. However, the effect of our device on physical and cognitive capabilities, which are crucial for well-being, and motivation for initiation and continuation of PA remains unclarified. In this study, we aimed to evaluate these effects. We hypothesized that our device may help improve capabilities, and that the enjoyment it provides may potentially contribute to increased motivation for initiation and continuation of PA. Fourteen healthy older adults participated in the study. Kickboxing lessons for the older adults using our device were conducted a total of five times over a six-week period, and the participants were free to attend the sessions as many times as they wished. They participated in three measurement sessions: pre-measurement before the lesson period, post-lesson measurement after each lesson, and post-measurement at the end of the lesson period to evaluate the effects of our device. Physical capabilities were assessed through two-step, stand-up, and center-of-pressure tests. Cognitive capabilities were assessed using illustration memory and computation tests. Mental state was assessed using a positive and negative affect schedule (PANAS). Subjective evaluations of enjoyment and motivation for the initiation and continuation of PA through the use of our device were also conducted. In the results, physical and cognitive capabilities and mental states tended to improve. Subjective evaluations indicated increased enjoyment from using our device and greater perceived effectiveness in motivating PA initiation and continuation. These evaluations improved over time. While the pre-post design without a control group limits causal interpretation, improvements in physical, cognitive, and mental states may have contributed to enhanced enjoyment. As a result, the device may have the potential to enhance motivation for initiation and continuation PA in older adults.

## 1 Introduction

Recently, the population of older adults aged 60 and over is growing rapidly (1). Since many older adults suffer from physical or mental health issues, rising medical and nursing care costs may pose significant economic burdens. Therefore, maintaining the health status of older adults is crucial to achieving a sustainable society, and the concept of healthy aging has been focused attracted attention and largely discussed. In 2015, the World Health Organization defined “healthy aging” as the process of developing and maintaining functional ability that enables well-being in older age (2). Functional ability is formed by the interaction of intrinsic capacities of older adults, which comprise their physical and mental capabilities, and environmental characteristics, including surroundings and support (3). Even if intrinsic capabilities decline with age, they can be compensated for with appropriate support and environment, helping to maintain well-being. However, it is imperative to preserve intrinsic capabilities to ensure their well-being.

There are numerous reports of intrinsic capabilities of older adults that are closely related to the continuation and promotion of their well-being. The relationship between physical capabilities and well-being has long been evident (4). A decline in grip strength, walking speed, and walking ability, which can be easily measured, is positively correlated with a decline in well-being (5–7). Cognitive capabilities are also closely related to well-being, with memory and executive functions particularly influencing well-being (8–10). Physical activity (PA) is crucial for maintaining these intrinsic capabilities and improving well-being. Unfortunately, many older adults lack PA (11). Physical and mental discomfort, lack of self-efficacy, poor body image, and time constraint can hinder initiating and continuing PA (12, 13), suggesting that PA is often not perceived as enjoyable. Additionally, activity restrictions such as staying at home and closure of sports facilities during the Covid-19 pandemic have decreased PA (14, 15).

One way to encourage more PA among older adults is through the use of technology. A recent comprehensive systematic review reported that digital exergames combined with PA and digital video games were effective in initiating and continuing PA in older adults at nursing homes (16). Other studies have also reported the effectiveness of digital exergames (17, 18), whose beneficial effects may be related to the positive emotional responses elicited through the games (19, 20). A method of integrating PA and music has been investigated as a way to elicit positive emotional responses (21). The method involves playing music through PA, and has been reported to extend exercise time in older adults. Interestingly, encouraging voluntary PA and self-selecting the frequency and duration of PA seems to have a high improvement effect on physical function (22). These methods are useful for addressing some factors that interfere with PA. However, several technical challenges remain in these methods. It has been noted that digital exercise games can be difficult to implement for older adults, as they require the selection of an appropriate computer platform and proper placement of sensors to measure body movements (23). There is also the possibility of boredom as the game often becomes monotonous. Methods that integrate music also require a lot of specialized equipment, which is a significant barrier for older adults.

In our previous studies, we attempted to address these challenges related to initiating and continuing PA. We focused on sensory augmentation, an approach that uses technology to extend or enhance human perception (24). We developed a boxing glove-type sensory augmentation device that used light, sound, and vibration to focus on visual, auditory, and tactile senses. The aim of our device is to perceive the improvement of physical movements through PA by enhancing these senses. This perception improves physical and mental comfort, self-efficacy, and body image, thereby making PA enjoyable and contributing to increased motivation for PA (25, 26). Our device is worn on the subject's hand, emitting sound, vibration, and light according to the subject's body movement. As the subject's punching motion improves, the production of sound, vibration, and light increases. Through this, we expect the subject to receive a positive image of exercise and improve self-efficacy, thereby eliciting greater enjoyment of PA. In our previous works, we also attempted to develop an enjoyable PA program using our device, incorporating kickboxing as the chosen form of PA. Studies involving young (23.3 ± 1.5 years) (25) and middle-aged (58.9 ± 17.1 years) (26) subjects demonstrated that the device increased positive emotions compared to the average in a survey of 1,500 Japanese people on their daily life post PA with our device. Additionally, 82% of the surveys subjects preferred and enjoyed using the device after using it for PA, and 69% of the subjects preferred it over regular boxing gloves. These results suggest that our device can make PA enjoyable and contribute to its continuation. However, the effect of our device on physical and cognitive capabilities, which are crucial for well-being, and motivation for initiation and continuation of PA remains unclarified.

The purpose of this study was to evaluate the effect of our device on physical and cognitive capabilities and motivation for initiation and continuation of PA as a preliminary study. We hypothesized that our device may help improve physical and cognitive capabilities, and that the enjoyment of our device potentially contributes to the motivation for initiation and continuation of PA.

## 2 Materials and method

### 2.1 Boxing glove-type sensory augmentation device

Human sensory augmentation was our primary focus, specifically auditory, tactile, and visual senses. We aimed to increase the information acquired by these sensory organs during PA in order to perceive the improvement of the body movements and to induce enjoyment (25, 26). To the best of our knowledge, our previous studies were the first to use sensory augmentation to elicit enjoyment during PA. Additionally, by focusing on kickboxing—a exercise that provides a high exercise effect observed in young individuals, characterized by many improvements such as upper-body muscle power, aerobic power, anaerobic fitness, flexibility, speed and agility even in short durations of about an hour (27) and similar exercise benefits have also been reported in older adults (28)—we aimed to maximize the benefits of PA with high efficiency in our previous studies (25, 26).

**Figure 1:**
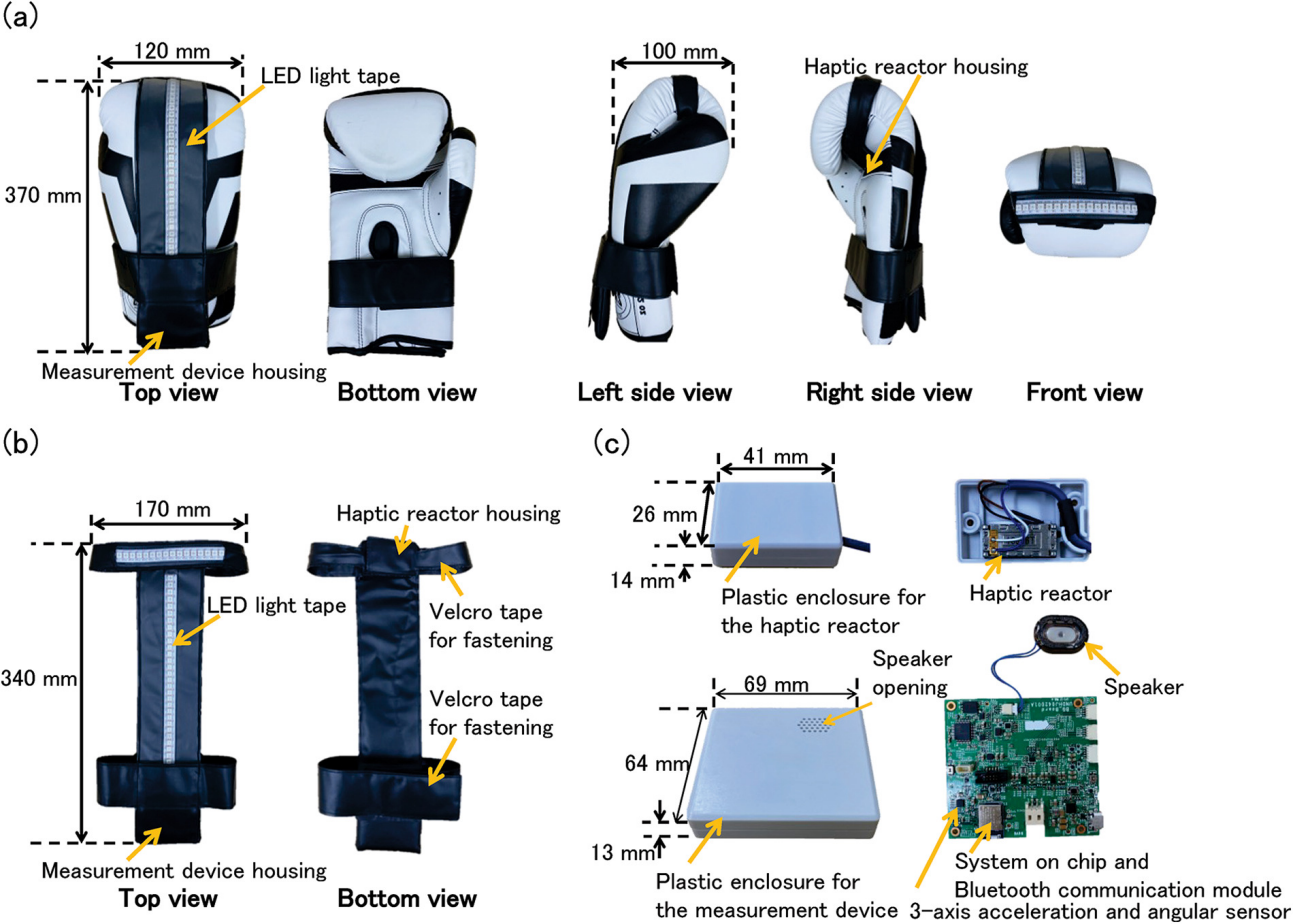
A picture of our boxing glove-type sensory augmentation device and its components. **(a)** Boxing glove with a belt equipped with LED light tape, measurement device housing, and haptic reactor housing. **(b)** Belt attached to the glove using Velcro tape. **(c)** Measurement device containing motion sensors, speaker, Bluetooth module, and battery; haptic reactor positioned in the finger area.

Figure 1a shows our boxing glove-type sensory augmentation device. This device consists of boxing glove and a belt equipped with a LED light tape (NeoPixel RGB TAPE LED, Peace corporation), housing for a measurement device, and housing for a haptic reactor (AFT14A903A, Alps Alpine). This belt is fastened to the boxing glove using Velcro tape, as shown in Figure 1b. As shown in Figure 1c, the measurement device has a plastic enclosure and mainly comprises a 3-axis acceleration and 3-axis angular sensor (ICM-20608, TDK), a speaker (P-12494, Akizukidenshi), and Bluetooth module (MDBT50Q-1MV2, Raytac) with an embedded system-on-chip (nRF52840, Nordic). The haptic reactor is enclosed in a plastic enclosure and positioned to align with the finger area of the boxing glove. This device is battery-powered and is also equipped with a lithium-polymer battery (LP602835, EEMB). This device is worn on the hand, augmenting the auditory, tactile, and visual senses using sound, vibration, and light stimuli based on the power and type of punch. Four different punches—jab, straight, hook, and uppercut—and their power are determined based on measured acceleration and angular velocity values. As the subject's punches improve, the sound, vibration, and light increases. This provides a positive exercise experience and satisfies self-efficacy, thereby eliciting enjoyment of PA. Additionally, measured acceleration, angular velocity, and punch determination results are displayed and recorded in real-time on an Android smartphone (Xiaomi Redmi Note 10, Xiaomi) to store these values and observe their changes using original software (Boxing Glove App) developed in our previous studies.

### 2.2 Subjects

Fourteen healthy older adults participated in this study, with mean age, height, and weight of 67.2 ± 5.8 years, 165.7 ± 10.1 cm, and 65.0 ± 13.7 kg, respectively. The criteria for older adults were set at 60 years of age and older (29). The subjects were recruited through local websites and flyers. People between the ages of 60 and 75 who were not restricted from PA by their doctors could apply as subjects. Exclusion criteria included individuals with high blood pressure or diabetes, those restricted from exercise by medical guidance, those who considered themselves unable to perform the PA in this study, and those with poor physical conditions (e.g., headache, fever, or other physical ailments). This study complied with the Declaration of Helsinki; informed consent was obtained from all participants, and they did not receive any monetary compensation. Additionally, this study was approved by the Ethics Review Committee for Medical and Health Research Involving Human Subjects (Natural Sciences) of Ritsumeikan University (BKC-LSMH-2023-032-1).

### 2.3 Experimental method

**Figure 2:**
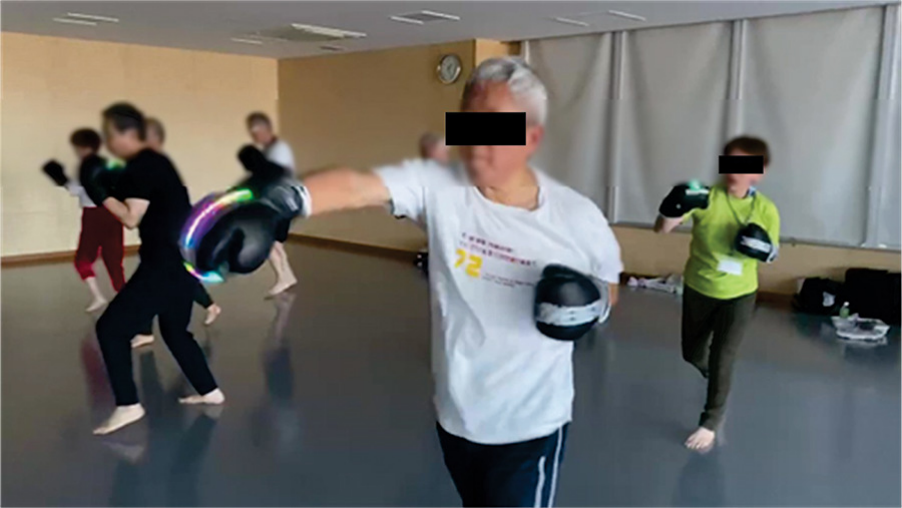
A picture of shadow boxing using a boxing glove-type sensory augmentation device.

**Figure 3:**
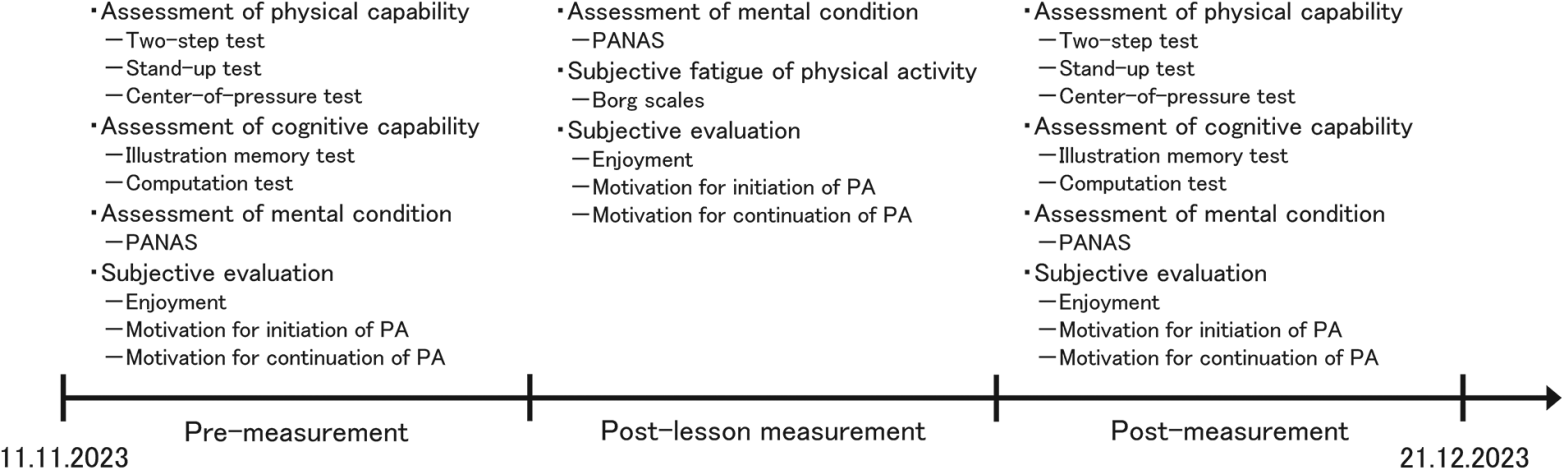
Flowchart of experiment in which the subjects participated.

Kickboxing lessons for the older adults were conducted a total of five times over a six-week period by Style Corporation, and participants were free to attend as many times as they wished. Lessons were held on November 11 and 25, and December 2, 8, and 21, 2023. Each 60-minutes lesson included preparatory exercises, shadow boxing using our device as shown in Figure 2, and mitts using regular boxing gloves. At first, subjects performed 20 min of full body stretching as a preparatory exercise. Then, after a 5 min break and time to wear the device, they performed shadow boxing for 25 min. A video of shadow boxing using our device has been included in the supplementary materials. Next, after a 5 min break and time to take off the device, they performed for 5 min with the mitts. Professional trainers accompanied the participants to ensure their safety and the proper implementation of the lessons. During this period, subjects participated in three different measurements: “pre-measurement” taken a few days before first lesson in which each subject participated, “post-lesson measurement” taken on the same day each lesson was completed, and “post-measurement” taken within a few days of the last lesson in which each subject participated at the end of the six-week period. As shown in Figure 3, physical and cognitive capabilities, mental states, and subjective evaluations about enjoyment and motivation for initiation and continuation of PA through the use of our device were assessed. In this study, physical and cognitive capabilities were assessed only “pre-measurement” and “post-measurement”. These assessments required approximately 60 min to complete, which would have significantly increased the burden on participants. Moreover, there were concerns that conducting assessments immediately after the kickboxing lessons could result in data being confounded by transient physical fatigue. Therefore, no assessments of physical and cognitive capabilities were performed during the post-lesson measurement; assessments were limited to pre-measurement and post-measurement. In pre-measurement, participants wore the device on their hand and experienced sound, vibration, and light according to their punches for the first time.

### 2.4 Measurements of physical capabilities, cognitive capabilities, and mental state

A two-step, stand-up, and center-of-pressure test were conducted in pre- and post-measurements. In the two-step test, subjects placed their toes on a starting line and walked two steps as large as possible. The distance to the toes of both feet together was measured, and the two-step value was calculated by dividing this distance by their height, which has been shown to correlate highly with results from other validated mobility assessments such as the 10-meter walk test, the 6-minute walk test, and the Timed Up and Go test (30, 31). These assessments are widely used to comprehensively evaluate walking ability in older adults (32, 33). Therefore, walking ability in the present study was assessed solely with this test. In the stand-up test, subjects stood up from a chair at different heights (40 cm and 30 cm) with either both or one foot grounded. We then assessed whether they could hold the standing position for 3 s (32). These two tests are common methods for evaluating physical capability, particularly lower limb function, and were developed by the Japanese Orthopedic Association (https://www.joa.or.jp/english/main.html) to assess difficulties in movement or performing daily activities due to age-related musculoskeletal disorders affecting muscles, joints, and bones. These methods are quick to administer and places minimal physical burden on older adults. The two-step test evaluates walking ability, such as leg muscle strength, balance, and flexibility, which decline with age. The standing test evaluates lower limb muscle strength, and success decreases with age. Additionally, the center-of-pressure in the standing still position for 30 s with eyes open was measured by a center-of-pressure meter (Stabilometer, T.K.K. 5810; SANKA Co., Ltd, Japan). The main specifications of this device are a load capacity of 10–120 kg, a measurement area of 360 mm × 360 mm, and a sampling frequency of 100 Hz. This device has been used to assess static balance capability in older adults (34, 35).

Illustration memory and computation tests were conducted in pre- and post-measurements. In the illustration memory test, subjects were asked to remember 16 illustrations, perform a computation test, and then recall the illustrations they remembered. These 16 illustrations were memorized by visually inspecting four A4 sheets each containing four printed illustrations. The time required for memorization was set at one minute per sheet. This test employed a cued recall procedure, which is used in the cognitive function test administered to drivers aged 75 and over in Japan as part of the license renewal process (36). This procedure is a method of assessing memory in older adults, wherein participants are asked to memorize specific illustrations, engage in an unrelated task, e.g., a computation test, and then recall the previously memorized items. This cognitive assessment has been shown to have high concordance with clinical diagnoses by physicians, including the Mini-Mental State Examination (MMSE) and the Hasegawa Dementia Scale-Revised, confirming its criterion-related validity (37). Furthermore, cognitive decline has been shown to negatively affect calculation ability, such as the time required to solve simple arithmetic problems and the accuracy of responses (38). This decline in numerical processing is reflected in widely used cognitive assessments such as the MMSE and the Montreal Cognitive Assessment, both of which include calculation tasks (39, 40). In the present study, we prepared and implemented an original calculation task to be performed during the interval between encoding and recall in the cued recall procedure, as we aimed to design a task with a time duration that would be appropriate for use within the illustration memory test. In this computation test, they were asked to solve 12 mixed arithmetic addition and subtraction questions. These questions were printed on an A4 size sheet, and the time required for computation was set at three minutes. The scores based on the correct answers were recorded, as scores tend to decrease with aging.

### 2.5 Questionnaires

A positive and negative affect schedule (PANAS), a self-report questionnaire consisting of two 10-item scales to measure both positive and negative affect (41), was conducted in the Japanese edition to assess mental states in all three measurements.

Subjective evaluations of “enjoyment,” “motivation for initiation of PA,” and “motivation for continuation of PA” through the use of our device were conducted using 5-point scales. In the questionnaire assessing enjoyment, participants were asked: “Did you enjoy using our boxing gloves?” In the questionnaire evaluating motivation, two items were presented: “Do you think our gloves are effective in promoting the initiation of PA?” and “Do you think our gloves are effective in supporting the continuation of PA?” These items were designed to assess participants' subjective perceptions regarding the motivational impact of our device. For each question, a score of “1” indicated high enjoyment, effectiveness in initiating PA, and effectiveness in continuing PA, while a score of “5” indicated low enjoyment, ineffectiveness in initiating PA, and ineffectiveness in continuing PA. Additionally, subjective fatigue during PA lessons was assessed using the Borg Scale (42).

### 2.6 Signal processing

We focused on the frequency component of the center-of-pressure to evaluate balance capability, based on the measurements shown in Section 2.4. While conventional indicators to evaluate the balance capability of older adults—such as the unfiltered root mean square amplitude, maximum amplitude, and total trajectory length calculated from measured center-of-pressure data without filtering —are useful (35, 34, 43), they do not capture the frequency characteristics of postural sway. Given that postural control involves multi-scale processes including biomechanical and neurophysiological mechanisms, frequency-domain analysis allows for a more detailed evaluation. For example, low- and mid-frequency components reflect central control of posture, as shifts in a moving reference point (44, 45), while high-frequency components reflect peripheral adjustments around that trajectory (44–46). In addition to these insights into the frequency components of postural control, it has been reported that the low-pass component (0.5 Hz) and band-pass components (0.5–5 Hz) of center-of-pressure are positively correlated with balance function, while the high-pass components (5–20 Hz) are negatively correlated (45). As balance capability declines with aging, low-pass and band-pass components of center-of-pressure decrease while the high-pass component increases. Therefore, these indicators were adapted to evaluate the balance capability of older adults.

In this study, we analyzed these frequency components of center-of-pressure in the anterior-posterior and medial-lateral directions. We initially downsampled the measured data, i.e., trajectory of the center of pressure, from 100 Hz to 20 Hz in order to retain the frequency range that appropriately reflects variations in center-of-pressure (47). Then, we applied a low-pass filter (0.5 Hz), band-pass filter (0.5–5 Hz), and high-pass filter (5 Hz) using a Butterworth filter (4th order, zero-phase lag). These filter settings were selected based on the methodology employed by Moreno et al. (45), who used a similar approach to investigate postural control through frequency-domain analysis. Subsequently, the maximum amplitude of the trajectory in the anterior-posterior and medial-lateral directions was computed to quantify the extent of postural sway, and we normalized these values by the subject's height. These signal processing operations were performed using MathWorks Matlab R2024b (version 24.2.0.2712019).

### 2.7 statistical analysis

The two-step values, three amplitudes of center-of-pressure, and scores of the illustration memory and computation tests were analyzed using the Wilcoxon signed-rank test. In cases where all measurement items during pre-lesson, post-lesson, and post-measurement could not be performed due to the subject's circumstances, the subject's data were excluded from statistical processing. This statistical analysis was conducted using IBM SPSS Statistic (version 29.0.0.0).

In the evaluation of the PANAS, the Shapiro–Wilk test was used to assess the normality of both positive and negative affect scores, in order to determine the appropriate statistical methods for data obtained at three time points: pre-, post-lesson, and post-measurement. This analysis was conducted using IBM SPSS Statistics (version 29.0.0.0). As a result, normality was rejected for the post-lesson measurement scores in negative affect group (*p* = 0.017). Therefore, the data were judged not to satisfy the assumption of normality, and non-parametric methods were selected for further analysis. Subsequently, a non-parametric repeated measures analysis was performed using the nparLD package in R. The LD-F1 model was applied to assess the effect of time (pre- post-lesson, and post-measurement) within each positive and negative affect group using Posit Software RStudio (version 2024.09.1 Build 394). When a significant difference was observed, pairwise comparisons were conducted using the Wilcoxon signed-rank test. Bonferroni correction was applied to adjust for multiple comparisons. The level of statistical significance was set at 5%. Additionally, relative treatment effects were calculated for each time point as non-parametric effect size indicators using the nparLD package.

In cases where all measurement items during pre-, post-lesson, and post-measurement could not be performed due to the subject's circumstances, the subject's data were excluded from these statistical processings.

## 3 Results

A total of five kickboxing lessons were conducted over a six-week period. The attendance of each participant is summarized in Table 1. Among the 14 participants, 13 attended at least one session, while one did not attend any. The number of sessions attended ranged from 0 to 5, with a median of 3. This variation likely reflects differences in individual availability to participate.

**Table 1:** Attendance record for each kickboxing session.

Subject ID	Lesson1	Lesson2	Lesson3	Lesson4	Lesson5	Sessions attended
1		✓		✓	✓	3
2	✓	✓	✓		✓	4
3	✓		✓	✓		3
4		✓	✓	✓	✓	4
5		✓			✓	2
6						0
7	✓	✓		✓		3
8	✓	✓		✓		3
9				✓	✓	2
10			✓	✓	✓	3
11		✓	✓		✓	3
12		✓	✓		✓	3
13				✓	✓	2
14		✓	✓	✓		3

**Figure 4:**
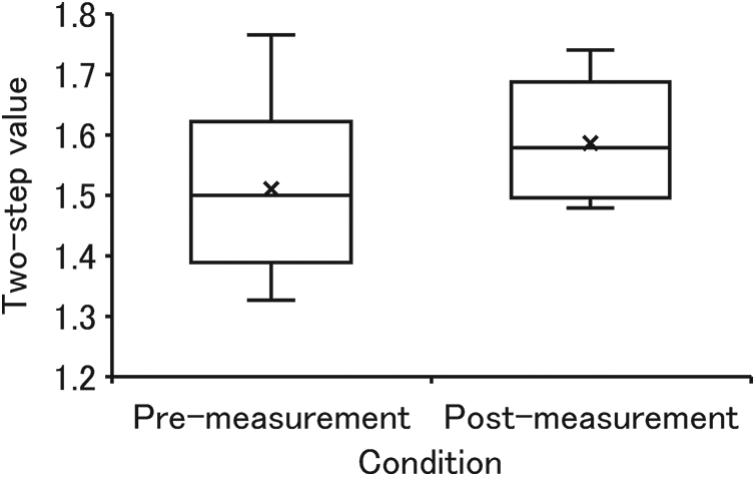
The two-step values measured in pre- and post-measurements.

In the physical capabilities test, four subjects were unable to participate in the measurements for personal reasons. Figure 4 shows the two-step value in pre- and post-measurements. The medians (Mdn) tended to increase between pre- (Mdn = 1.50) and post-measurements (Mdn = 1.58), with no significant difference identified (Z = 1.602, *p* = 0.109). The effect size was large (r = 0.507). As shown in Table 2, all subjects successfully stood up on both legs in all measurements. The number of subjects who successfully stood up at 40 cm increased from eight to ten, and at 30 cm, it increased from four to five.

**Table 2:** Number of successful participants.

Stand up condition	Pre-measurement	Post-measurement
Both legs; standing up from 40 cm	10	10
One leg; standing up from 40 cm	8	10
Both legs; standing up from 30 cm	10	10
One leg; standing up from 30 cm	4	5

The median, first and third quartile of center-of-pressure amplitude were shown in Table 3. The center-of-pressure amplitude at the anterior-posterior direction of the low-pass component tended to increase between pre- and post-measurements, with no significant difference found (Z = 1.478, *p* = 0.139). The effect size was medium (r = 0.467). One of the band-pass components also tended to increase between pre- and post-measurements, with no significant difference identified (Z = 0.968, *p* = 0.333). The effect size was medium (r = 0.306). One of the high-pass components tended to decrease between pre- and post-measurements, with no significant difference identified (Z = −0.357, *p* = 0.721). The effect size was small (r = 0.113). Additionally, the center-of-pressure amplitude at the medial-lateral direction of the low-pass component tended to increase between pre- and post-measurements, with no significant difference found (Z = 1.274, *p* = 0.203). The effect size was medium (r = 0.403). One of the band-pass components also tended to decrease between pre- and post-measurements, with no significant difference identified (Z = 0.153, *p* = 0.878). The effect size was very small (r = 0.048). One of the high-pass components tended to decrease between pre- and post-measurements, with no significant difference identified (Z = −1.478, *p* = 0.139). The effect size was medium (r = 0.467).

**Table 3:** Center-of-ptrssure amplitudes in pre- and post-measurement.

Center-of-ptrssure direction	Frequency components	Pre-measurement	Post-measurement
Anterior-posterior	Low-pass	0.57 (0.47, 0.75)	0.70 (0.57, 0.88)
	Band-pass	0.34 (0.31, 0.42)	0.41 (0.33, 0.63)
	High-pass	0.10 (0.05, 0.14)	0.07 (0.05, 0.17)
Medial-lateral	Low-pass	0.80 (0.73, 1.01)	1.31 (0.90, 1.53)
	Band-pass	0.50 (0.36, 0.59)	0.46 (0.40, 0.50)
	High-pass	0.15 (0.08, 0.26)	0.11 (0.08, 0.13)

**Figure 5:**
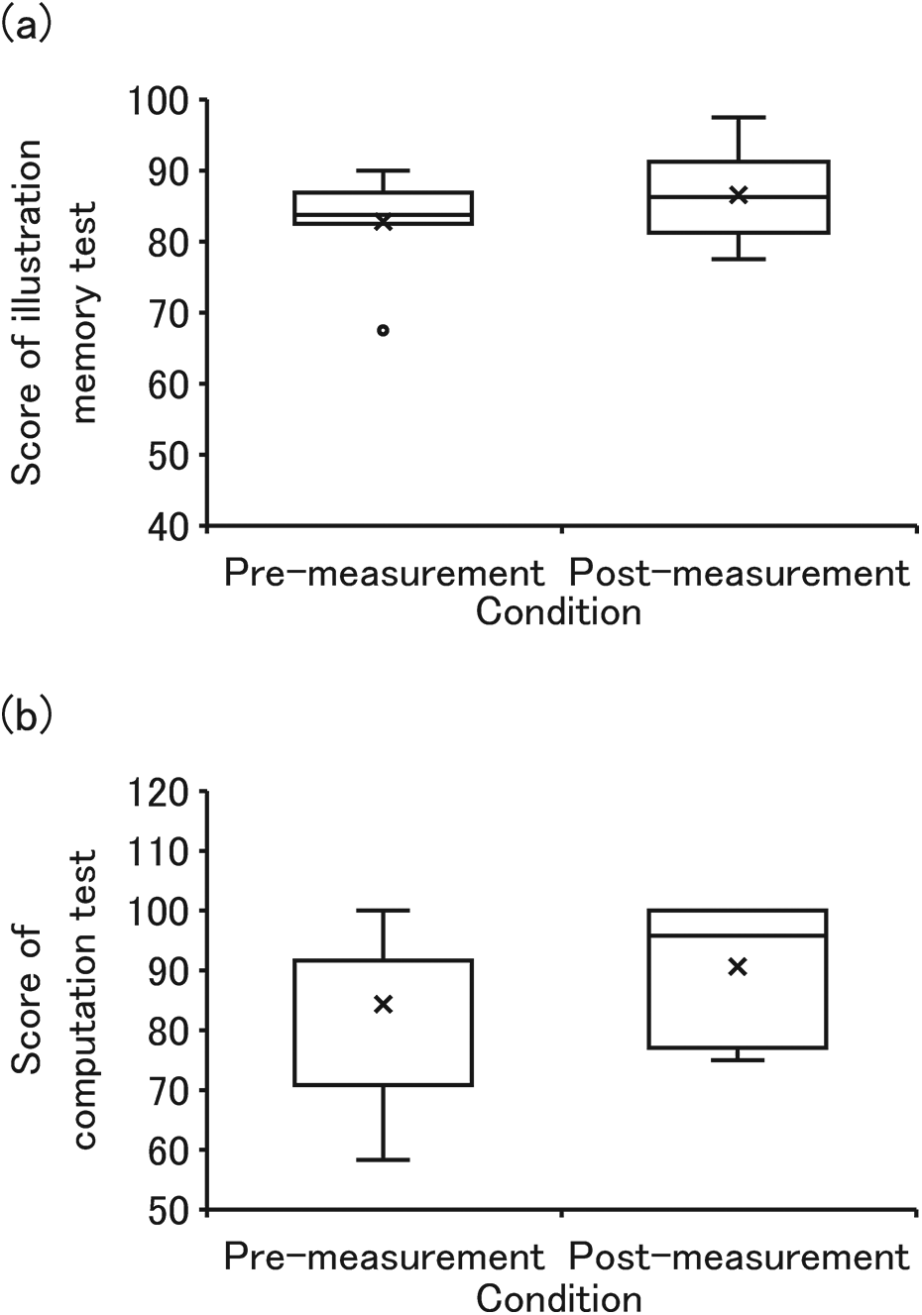
The scores of illustration memory test and computation test. **(a,b)** are scores of illustration memory test and computation test.

In the cognitive capabilities test, six subjects were unable to participate in the measurements due to personal reasons. Figure 5a displays the scores in the illustration memory test. An improvement in median value was observed between pre- (Mdn = 83.9) and post-measurement (Mdn = 86.3), with no significant differences identified (Z = 0.207, *p* = 0.207). The effect size was medium (r = 0.446). Figure 5b shows the scores in the computation test. Similarly, an improvement in median value between pre- (Mdn = 91.7) and post-measurement (Mdn = 95.8) was observed, but no significant differences were identified (Z = 1.035, *p* = 0.301). The effect size was medium (r = 0.366).

**Figure 6:**
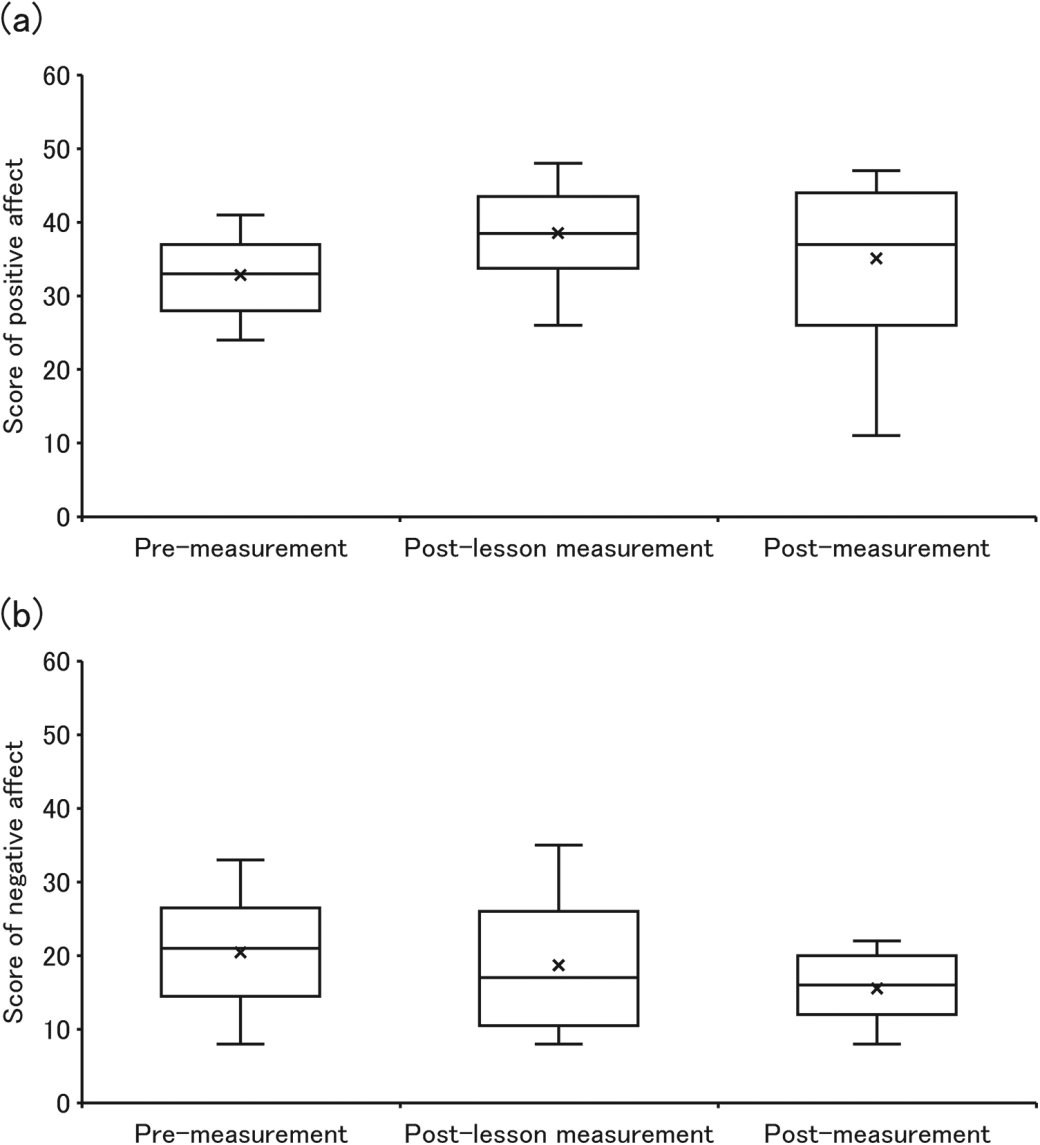
The scores of both positive and negative affect on PANAS. **(a,b)** are “score of positive affect” and “score of negative affect”.

Figure 6 displays the scores of both positive and negative affect in PANAS for pre-measurement (*n* = 13), post-lesson measurement (*n* = 34), and post-measurement (*n* = 11). No significant differences were found in positive affect scores across pre-, post-lesson, and post-measurement (ATS = 2.864, *p* = 0.071). However, there was a trend toward increased scores across the three time points (Mdn = 33.0, 38.2, and 35.0). The relative treatment effects were 0.372 at pre-measurement, 0.606 at post-lesson measurement, and 0.522 at post measurement, indicating a nonparametric trend toward increased positive affect following the post-lesson measurement, with partial retention at the post-measurement. Similarly, no significant differences were observed in negative affect scores across pre-, post-lesson, and post-measurement (ATS = 1.665, *p* = 0.193). Nonetheless, the median scores showed a decreasing trend (Mdn = 21.0, 17.0, and 16.0), and the relative treatment effects were 0.570 at pre-measurement, 0.510 at post-lesson measurement, and 0.420 at post-measurement, suggesting a nonparametric trend toward reduced negative affect following the post-lesson and post-measurement.

The average value of subjective fatigue after the lessons was 16.4 ± 2.1, indicating an exercise intensity equivalent to “hard” to “very, very hard” (42). Response ratio (%) of subjective evaluations of “enjoyment,” “motivation for initiation of PA,” and “motivation for continuation of PA” through the use of our device are shown in Tables 4–6 respectively. Most subjects indicated scores of 1, indicating “enjoyable,” for subjective evaluations of “enjoyment,” with an increase in the number of times. Similarly, many indicated scores of 1, indicating effectiveness in initiating and continuing PA, for subjective evaluations of “motivation for initiation of PA” and “motivation for continuation of PA,” with an increase in the number of times.

**Table 4:** Response rate (%) of subjective evaluation “enjoyment”.

Subjective evaluation “enjoyment”	Pre-measurement	Post-lesson measurement	Post-measurement
1	50.0	76.7	90.9
2	50.0	20.0	9.1
3	0.0	0.0	0.0
4	0.0	0.0	0.0
5	0.0	3.3	0.0

**Table 5:** Response rate (%) of subjective evaluation “motivation for initiation of PA”.

Subjective evaluation “motivation for initiation of PA”	Pre-measurement	Post-measurement
1	33.3	45.5
2	58.3	45.5
3	8.3	9.1
4	0.0	0.0
5	0.0	0.0

**Table 6:** Response rate (%) of subjective evaluation “motivation for continuation of PA”.

Subjective evaluation “motivation for continuation of PA”	Pre-measurement	Post-measurement
1	33.3	54.5
2	25.0	36.4
3	41.7	9.1
4	0.0	0.0
5	0.0	0.0

## 4 Discussion

The purpose of the study reported in this paper was to evaluate the effect of our device on physical and cognitive capabilities and motivation for initiation and continuation of PA in older adults. We hypothesized that our device may help improve physical and cognitive capabilities, and that the enjoyment of our device potentially contributes to the motivation for initiation and continuation of PA. In the verification experiment, we assessed physical capabilities using a two-step, stand-up, and center-of-pressure test, and cognitive capabilities using an illustration memory and computation test. Additionally, we assessed mental states using PANAS. Subjective evaluation based on a five-point rating scale questionnaire regarding enjoyment and motivation for initiation and continuation of PA through the use of our device were also assessed.

We observed that the two-step value tended to increase from pre- to post-measurement with a large effect size. The two-step is an indicator for evaluating the walking ability of the older adults, and tends to decline with age (32, 48). When the value is 1.3 or less, it indicates the beginning of a decline in mobility. Functional improvement through PA training has been reported and recommended (49). None of the older adults who participated in this study met this criterion, meaning that they did not experience a serious decline in mobility due to aging. In addition, after measurement, the trend of increasing two-step values may indicate that mobility is improving as a physical function. The stand-up test is an indicator that evaluates the lower limb muscle strength of the older adults, and also tends to decline with age (48). If a person is unable to stand up on one leg from a height of 40 cm, it indicates the beginning of a decline in walking ability. Functional improvement through PA training has been demonstrated and recommended (50). In this experiment, two people fell below this criterion before the PA, while after the PA all of them cleared this criterion; hence, the decline in mobility was reduced. Therefore, our results suggest that participating in this experiment may have contributed to improvement in physical capabilities in older adults.

Additionally, the low-pass components of the center-of-pressure amplitudes in both the anterior-posterior and medial-lateral directions showed increasing trends in post-measurement. The band-pass component in the anterior-posterior direction also showed a trend toward increase in post-measurement. The high-pass component in both the anterior-posterior and medial-lateral directions tended to decrease. Based on previous studies (45), our findings suggest improvements in balance ability. In summary, in this experiment, no significant difference was observed between pre- and post-measurement in terms of physical capability indicators. This is thought to be due to the small number of research subjects and the low frequency of PA. However, an improvement trend was observed in many of the physical ability assessments. The effect size in these tests tended to be large to moderate. Therefore, these results suggest that participating in this experiment may have contributed to improvement in the physical capabilities in older adults, particularly mobility and balance.

The median scores of the illustration memory and computation tests tended to increase from pre-measurement to post-measurement. As memory function is a cognitive capability, it decreases with age. This test is used to test the cognitive capability when older adults (over 75 years old) drivers renew their driver's license in Japan. The computation test reflects executive function, a cognitive function that declines with age. In other words, improvement in cognitive capabilities increases with scores of illustration memory and computation tests. In summary, as shown in Figure 5, no significant differences were identified between pre- and post- measurement. Although this was also possibly due to the short duration and low frequency of PA, a trend toward improvement in all cognitive capability assessments was observed. As it is well known that physical activity activates brain activity (51), it may be considered that participating in this experiment may have contributed to improvement in cognitive capabilities.

In the positive affect of PANAS, we observed a tendency for substantial changes from pre-measurement to post-lesson measurement and from post-lesson measurement to post-measurement. That is, the median scores were 33.0 at pre-measurement, 38.2 at post-lesson measurement, and 35.0 at post-measurement, showing an increase after the post-lesson measurement with a slight decline at the follow-up. Correspondingly, the relative treatment effects were 0.372 at pre-measurement, 0.606 at post-lesson measurement, and 0.522 at post-measurement indicating a notable nonparametric increase in positive affect following the post-lesson measurement. Although there was a slight reduction at post-measurement, the relative treatment effect remained higher than at pre-measurement, suggesting that the improvement was at least partially sustained. Such increase from pre-measurement to post-lesson measurement through use of our enjoyable device was similar to that in our previous study (25, 26). It can be considered that participating in this experiment may enhance positive affect in older adults. In other words, the fact that we were able to clearly recognize the movement of the body and its state through sensory extension of sight, hearing and touch suggests that it is possible to make exercise fun for the older adults as well.

Negative affect showed a tendency to decrease across pre-, post-lesson, and post-measurement. The median scores declined from 21.0 at pre-measurement to 17.0 at post-lesson measurement and further to 16.0 at post-lesson measurement, and the corresponding relative treatment effects were 0.570, 0.510, and 0.420, respectively. Despite the subjective fatigue level in this experiment being 16.4 ± 2.1, indicating high exercise intensity, i.e., exercise that causes physical discomfort, negative affect scores continued to trend downwards. This indicates that discomfort associated with PA was mitigated. Pleasurable experiences during PA and intrinsic motivation to engage in PA are known to reduce discomfort (13), suggesting that participating in this experiment may facilitate these effects and reduce negative affect associated with PA. Previous studies have shown that reducing PA discomfort through activities like listening to music or watching videos supports sustained PA (20, 21), likely due in part to the release of endorphins in the brain Endorphins are neurotransmitters that alleviate pain and stress (21, 52). It is thought that these are related to the distraction of consciousness from the pain of physical exercise and increase in pain tolerance associated with increase in endorphin levels. In this study, it is thought that participating in this experiment may promote internal motivation by enhancing pleasurable experiences during PA, and that fun PA caused a distraction of consciousness and an increase in endorphin secretion.

As shown in Table 4 numerous subjects indicated ratings of 1, indicating “enjoyable” in their subjective evaluations of enjoyment through the use of our device. This trend was evident even during pre-measurement, before the kickboxing lesson, suggesting that our device may be effective at inducing enjoyment in this experiment, similar to our previous studies (25, 26). Even before the lesson using our device, the fact that they were able to find enjoyment was probably because they were able to clearly recognize their own body movements through the expansion of their sense of sight, hearing, and touch. Furthermore, these positive evaluations increased in both the post-lesson measurement and post-measurement sessions. This suggests that more frequent participation in PA using our device may be associated with greater enjoyment. This may be due to the fact that using our device allows one to recognize changes, not just physical movement. In addition, as shown in Tables 5, 6, the number of people who answered “1” (i.e., “effective”) in the subjective evaluation of the motivation for initiation and continuation of PA through the use of our device increased. In other words, it can be considered that the participants recognized the use of our device as effective for initiation and continuation of PA. Based on the intended purpose of our device, it can be considered that the enjoyment of PA induced by our device led to such changes in motivation. From this, it can be thought that our device could potentially contribute to the motivation of the older adults regarding PA.

Although caution is required due to the pre-post comparison design without a control group —i.e., the effects of the device and those of PA alone may be confounded—physical and cognitive capabilities showed a tendency toward improvement. In other words, it can be assumed that the older ages were able to clearly recognize their own progress in PA. This kind of recognition may help alleviate physical and mental discomfort, low self-efficacy, and poor body image—factors that make it difficult to continue PA, thereby inducing enjoyment. Among these, improving self-efficacy is particularly important. Older adults with a high sense of self-efficacy have a high motivation for exercise (53). In addition, this contributes to maintaining and improving adherence to exercise programs (54, 55), and is thought to have led to increased motivation in this study. As PA is known to improve physical and cognitive capabilities on its own (51, 56), the observed improvements of physical and cognitive capabilities, and mental status may reflect a combination of general PA effects and device-induced augmentation, rather than the device alone. Although it is difficult to disentangle these effects in this study, subjective evaluations suggest that our device may contribute to enhancing enjoyment, motivation for initiation and continuation of PA. Moreover, it is also possible that our device played a role in the observed improvements in physical and cognitive functions.

## 5 Limitations

It should be noted that the results of this study are based on a relatively small sample size. In addition, the study is limited by the absence of a comparison group—that is, a group performing physical activity (PA) without the use of our device. In other words, it is difficult to determine whether the observed improvements in motivation to initiate and continue PA were due to the device itself or to the combination of the device and physical activity.

Additionally, information regarding the prior PA of the participants with martial arts or competitive sports, as well as their blood pressure and lung capacity, was not collected. However, this omission is not expected to have significantly influenced the outcomes, as the participant group was relatively homogeneous and randomly assigned.

## 6 Conclusion

In this study, we evaluated the effects of our device developed in our previous research, on the physical and cognitive capabilities and motivation for initiation and continuation of PA among older adults. Subjective evaluations regarding the enjoyment of our device indicated that participants perceived it as enjoyable, which may suggest that the observed improvements in physical and cognitive capabilities contributed to this perception. This sense of enjoyment may have led participants to perceive the device as effective in promoting the initiation and continuation of PA. However, as this study employed a pre-post comparison design without a control group, it is difficult to demonstrate the definitive effects of our device. Nonetheless, the observed improvements in subjective motivation suggest that the device may play a role in encouraging PA participation among older adults. Based on these findings, our device can be considered one contributing factor in supporting motivation and continued engagement in PA among older adults. In the future, for the practical application of our device, it will be necessary to increase the number of older adults and conduct large-scale experiments that include a comparison group.

Our device requires no special equipment. Therefore, it can be used in existing PA lesson at sports facilities, as in this experiment, or at the subject's home. Although we focused on kickboxing as a PA that can be effective in a short time, our sensory augmentation device for auditory, tactile, and visual sensations can be applied to other PA. This is expected to promote PA during leisure time and contribute significantly to older adults' well-being and healthy aging.

## Data Availability

The datasets presented in this article are not readily available because the data that support the findings of this study are not publicly available due to privacy or ethical restrictions. The data include personal information, and even anonymized data cannot be shared freely as per the regulations of the ethics committee. Researchers who wish to access the data for specific purposes may contact the corresponding author, subject to approval by the ethics committee. Requests to access the datasets should be directed to Yusuke Sakaue, sakaue@fc.ritsumei.ac.jp.

## References

[1] He W Goodkind D Kowal P. An Aging World: 2015. International Population Reports. Washington, DC: U.S. Government Publishing Office (2016).

[2] Michel JP Sadana R. "Healthy aging" concepts and measures. J Am Med Dir Assoc (2017) 18(6):460–4. 10.1016/j.jamda.2017.03.008 28479271

[3] Fallon CK Karlawish J. Is the WHO definition of health aging well? Frameworks for "health" after three score and ten. Am J Public Health. (2019) 109(8):1104–6. 10.2105/AJPH.2019.305177 31268759 PMC6611105

[4] Wilson IB Cleary PD. Linking clinical variables with health-related quality of life. A conceptual model of patient outcomes. JAMA. (1995) 273(1):59–65. 10.1001/jama.1995.03520250075037 7996652

[5] Xie H Lu S. The association between physical performance and subjective wellbeing in Chinese older adults: a cross-sectional study. Front Public Health. (2022) 10:965460. 10.3389/fpubh.2022.965460 36187619 PMC9521645

[6] Minghetti A Donath L Hanssen H Roth R Lichtenstein E Zahner L Physical performance, cardiovascular health and psychosocial wellbeing in older adults compared to oldest-old residential seniors. Int J Environ Res Public Health. (2022) 19(3):1451. 10.3390/ijerph19031451 35162467 PMC8835371

[7] Saadeh M Xia X Verspoor E Welmer AK Dekhtyar S Vetrano DL Trajectories of physical function and behavioral, psychological, and social well-being in a cohort of Swedish older adults. Innov Aging. (2023) 7(5):igad040. 10.1093/geroni/igad040 37360217 PMC10287187

[8] Wilson RS Boyle PA Segawa E Yu L Begeny CT Anagnos SE The influence of cognitive decline on well-being in old age. Psychol Aging. (2013) 28(2):304–13. 10.1037/a0031196 23421323 PMC3692607

[9] Ihle A Oris M Sauter J Spini D Spini D Rimmele U The relation of low cognitive abilities to low well-being in old age is attenuated in individuals with greater cognitive reserve and greater social capital accumulated over the life course. Aging Ment Health. (2020) 24(3):387–94. 10.1080/13607863.2018.1531370 30588833

[10] Fastame MC. Are subjective cognitive complaints associated with executive functions and mental health of older adults? Cogn Process. (2022) 23(3):503–12. 10.1007/s10339-022-01089-y 35380282 PMC9296425

[11] World Health Organization. Global Status Report on Physical Activity 2022. Geneva, Switzerland: World Health Organization (2022).

[12] Williams NH Hendry M France B Lewis R Wilkinson C. Effectiveness of exercise-referral schemes to promote physical activity in adults: systematic review. Br J Gen Pract. (2007) 57(545):979–86. 10.3399/096016407782604866 18252074 PMC2084138

[13] Ekkekakis P Parfitt G Petruzzello SJ. The pleasure and displeasure people feel when they exercise at different intensities: decennial update and progress towards a tripartite rationale for exercise intensity prescription. Sports Med. (2011) 41(8):641–71. 10.2165/11590680-000000000-00000 21780850

[14] Woods JA Hutchinson NT Powers SK Roberts WO Gomez-Cabrera MC Radak Z The COVID-19 pandemic and physical activity. Sports Med Health Sci. (2020) 2:55–64. 10.1016/j.smhs.2020.05.006 34189484 PMC7261095

[15] Naveed N Ahmad K Majeed H Qureshi K Ahmad I Awan MF The global impact of COVID-19: a comprehensive analysis of its effects on various aspects of life. Toxicol Res. (2024) 13(2):1–13. 10.1093/toxres/tfae045 PMC10964844 38545435

[16] Diener J Rayling S Bezold J Krell-Roesch J Woll A Wunsch K. Effectiveness and acceptability of e- and m-health interventions to promote physical activity and prevent falls in nursing homes—a systematic review. Front Physiol. (2022) 13:894397. 10.3389/fphys.2022.894397 35669573 PMC9163679

[17] Bock BC Dunsiger SI Ciccolo JT Serber ER Wu WC Tilkemeier P Exercise videogames, physical activity, and health: wii heart fitness: a randomized clinical trial. Am J Prev Med. (2019) 56(4):501–11. 10.1016/j.amepre.2018.11.026 30777705 PMC7100962

[18] Anders P Bengtson EI Grønvik KB Skjæret-Maroni N Vereijken B. Balance training in older adults using exergames: game speed and cognitive elements affect how seniors play. Front Sports Act Living. (2020) 2:54. 10.3389/fspor.2020.00054 33345045 PMC7739609

[19] Glen K Eston R Loetscher T Parfitt G. Exergaming: feels good despite working harder. PLoS One. (2017) 12(10):e0186526. 10.1371/journal.pone.0186526 29059227 PMC5653295

[20] Bock BC Palitsky R Dunsiger SI Williams DM Serber ER. Exercise video games are associated with more positive affective response, which predicts physical activity adherence. Psychol Sport Exerc. (2021) 52:101802. 10.1016/j.psychsport.2020.101802 37975018 PMC10653676

[21] Rehfeld K Fritz TH Prinz A Schneider L Villringer A Witte K. Musical feedback system Jymmin® leads to enhanced physical endurance in the elderly—a feasibility study. Front Sports Act Living. (2022) 4:915926. 10.3389/fspor.2022.915926 36032261 PMC9403307

[22] Jungreitmayr S Kranzinger C Venek V Ring-Dimitriou S. Effects of an app-based physical exercise program on selected parameters of physical fitness of females in retirement: a randomized controlled trial. Front Physiol. (2022) 13:821773. 10.3389/fphys.2022.821773 35317213 PMC8934397

[23] Smeddinck JD Herrlich M Wang X Zhang G Malaka R. Work hard, play hard: how linking rewards in games to prior exercise performance improves motivation and exercise intensity. Entertain Comput. (2019) 29:20–30. 10.1016/j.entcom.2018.10.001

[24] Macpherson F. Sensory substitution and augmentation: an Introduction. In: Macpherson F, editor. Proceeding of the British Academy. Oxford: Oxford University Press (2018). p. 1–42. 10.5871/bacad/9780197266441.003.0001

[25] Kondo Y Okada S Saito M Tanaka T Fumimoto K. Development of boxing glove type sensation device for exercise induction and continuation. In Pro. IEEE 11th Glob. Conf. Cons. Elec. (GCCE). Osaka: IEEE (2022). p. 509–10. 10.1109/GCCE56475.2022.10014049

[26] Kondo Y Okada S Manno M Sakaue Y Makikawa M. Verification of the effects of exercise on the body and mind using a boxing glove-type sensory augmentation device. AHFE Int. (2023) 113:391–7. 10.54941/ahfe1004212

[27] Ouergui I Hssin N Haddad M Padulo J Franchini E Gmada N The effects of five weeks of kickboxing training on physical fitness. Muscles Ligaments Tendons J. (2014) 4(2):106–13. 25332919 PMC4187584

[28] Valdés-Badilla P Herrera-Valenzuela T Ramirez-Campillo R Aedo-Muñoz E Báez-San Martín E Ojeda-Aravena A Effects of Olympic combat sports on older Adults’ health Status: a systematic review. Int J Environ Res Public Health. (2021) 18(14):7381. 10.3390/ijerph18147381 34299833 PMC8303637

[29] United Nations. World Population Aging 2017 report (2017).

[30] Matsunaga S. Development of a convenient way to predict ability to walk, using a two-step test. J Showa Med Assoc. (2003) 63(3):301–8. 10.14930/jsma1939.63.301

[31] Kojima K Kamai D Ishitani S Watanabe S. Availability of the two-step test to evaluate balance in frail people in a day care service. J Phys Ther Sci. (2017) 29:1025–8. 10.1589/jpts.29.1025 28626315 PMC5468190

[32] Yoshimura N Muraki S Oka H Tanaka S Ogata T Kawaguchi H Association between new indices in the locomotive syndrome risk test and decline in mobility: third survey of the ROAD study. J Orthop Sci. (2015) 20(5):896–905. 10.1007/s00776-015-0741-5. Erratum in: *J Orthop Sci*. 20(5), 906. doi: 10.1007/s00776-015-0765-x. 26104219 PMC4575347

[33] Miyashita T Kudo S Maekawa Y. Assessment of walking disorder in community-dwelling Japanese middle-aged and elderly women using an inertial sensor. PeerJ. (2021) 9:e11269. 10.7717/peerj.11269 33954059 PMC8052961

[34] Katsura Y Takeda N Hara T Takahashi S Nosaka K. Comparison between eccentric and concentric resistance exercise training without equipment for changes in muscle strength and functional fitness of older adults. Eur J Appl Physiol. (2019) 119(7):1581–90. 10.1007/s00421-019-04147-0 31055678

[35] Yamazaki K Sakai Y Ito T Fukuhara J Morita Y. Percentage of decline in individual proprioceptors in older adults. J Phys Ther Sci. (2024) 36(9):492–7. 10.1589/jpts.36.492 39239420 PMC11374179

[36] National Police Agency. Cognitive Assessment (n.d.). Available online at: https://www.npa.go.jp/policies/application/license_renewal/ninti/index2.htm (Accessed June 21, 2025).

[37] National Police Agency. Survey on the relationship between cognitive function and safe driving: A research project in response to the proposal for preventing traffic accidents involving elderly drivers [in Japanese] (2016). Available online at: https://www.npa.go.jp/koutsuu/kikaku/koureiunten/menkyoseido-bunkakai/cognitivef/cognitivef_report.pdf (Accessed June 21, 2025).

[38] Lemaire P Arnaud L. Young and older adults’ strategies in complex arithmetic. Am J Psychol Spring. (2008) 121(1):1–16. 10.2307/20445440 18437798

[39] Folstein MF Folstein SE McHugh PR. “Mini-mental state”: a practical method for grading the cognitive state of patients for the clinician. J Psychiatr Res. (1975) 12:189–98. 10.1016/0022-3956(75)90026-6 1202204

[40] Nasreddine ZS Phillips NA Bédirian V Charbonneau S Whitehead V Collin I The Montreal cognitive assessment, MoCA: a brief screening tool for mild cognitive impairment. J Am Geriatr Soc. (2005) 53:695–9. 10.1111/j.1532-5415.2005.53221.x 15817019

[41] Watson D Clark LA Tellegen A. Development and validation of brief measures of positive and negative affect: the PANAS scales. J Pers Soc Psychol. (1998) 54(6):1063–70. 10.1037//0022-3514.54.6.1063 3397865

[42] Borg GA. Psychophysical bases of perceived exertion. Med Sci Sports Exerc. (1982) 14(5):377–81. 10.1249/00005768-198205000-00012 7154893

[43] Quijoux F Nicolaï A Chairi I Bargiotas I Ricard D Yelnik A A review of center of pressure (COP) variables to quantify standing balance in elderly people: algorithms and open-access code. Physiol Rep. (2021) 9:e15067. 10.14814/phy2.15067 34826208 PMC8623280

[44] Zatsiorsky VM Duarte M. Instant equilibrium point and its migration in standing tasks: rambling and trembling components of the stabilogram. Motor Control. (1999) 3(1):28–38. 10.1123/mcj.3.1.28 9924099

[45] Moreno FJ Caballero C Barbado D. Postural control strategies are revealed by the complexity of fractional components of COP. J Neurophysiol. (2022) 127(5):1289–97. 10.1152/jn.00426.2021 35353616

[46] Caballero Sánchez C Barbado Murillo D Davids K Moreno Hernández FJ. Variations in task constraints shape emergent performance outcomes and complexity levels in balancing. Exp Brain Res. (2016) 234:1611–22. 10.1007/s00221-016-4563-2 26838357

[47] Caballero C Barbado D Moreno FJ. Non-linear tools and methodological concerns measuring human movement variability: an overview. Eur J Hum Mov. (2014) 32:61–81.

[48] Nakamura K Ogata T. Locomotive syndrome: definition and management. Clin Rev Bone Miner Metab. (2016) 14(2):56–67. 10.1007/s12018-016-9208-2 27375370 PMC4906066

[49] Matsushita D Gu Y. Improving locomotive syndrome risk level through community-led activities to establish walking habits. Jpn Archit Rev. (2024) 7:e12433. 10.1002/2475-8876.12433

[50] Aoki K Sakuma M Ogisho N Nakamura K Chosa E Endo N. The effects of self-directed home exercise with serial telephone contacts on physical functions and quality of life in elderly people at high risk of locomotor dysfunction. Acta Med Okayama. (2015) 69(4):245–53. 10.18926/AMO/53561 26289916

[51] Bliss ES Biki SM Wong RHX Howe PRC Mills DE. The benefits of regular aerobic exercise training on cerebrovascular function and cognition in older adults. Eur J Appl Physiol. (2023) 123:1323–42. 10.1007/s00421-023-05154-y 36801969 PMC9938957

[52] Fritz TH Bowling DL Contier O Grant J Schneider L Lederer A Musical agency during physical exercise decreases pain. Front Psychol. (2018) 8:2312. 10.3389/fpsyg.2017.02312 29387030 PMC5776142

[53] Colcombe S Kramer AF. Fitness effects on the cognitive function of older adults: a meta-analytic study. Psychol Sci. (2003) 14(2):125–30. 10.1111/1467-9280.t01-1-01430 12661673

[54] Sullivan AN Lachman ME. Behavior change with fitness technology in sedentary adults: a review of the evidence for increasing physical activity. Front Public Health. (2017) 4:289. 10.3389/fpubh.2016.00289 28123997 PMC5225122

[55] Castillo-Mayén R Cano-Espejo C Luque B Cuadrado E Gutiérrez-Domingo T Arenas A Influence of self-efficacy and motivation to follow a healthy diet on life satisfaction of patients with cardiovascular disease: a longitudinal study. Nutrients. (2020) 12(7):1903. 10.3390/nu12071903 32605026 PMC7400119

[56] Valenzuela PL Saco-Ledo G Morales JS Gallardo-Gómez D Morales-Palomo F López-Ortiz S Effects of physical exercise on physical function in older adults in residential care: a systematic review and network meta-analysis of randomised controlled trials. Lancet Healthy Longev. (2023) 4(6):e247–56. 10.1016/S2666-7568(23)00057-0 37182530

